# Adolescent utilization of eating disorder higher level of care: roles of family-based treatment adherence and demographic factors

**DOI:** 10.1186/s40337-024-00976-3

**Published:** 2024-02-02

**Authors:** Josephine S. Lau, Andrea H. Kline-Simon, Julie A. Schmittdiel, Stacy A. Sterling

**Affiliations:** 1 Department of Adolescent Medicine, The Permanente Medical Group, Kaiser Permanente Northern California, Oakland, USA; 2 Division of Research, Kaiser Permanente Northern California, Oakland, USA; 3 Department of Health System Science, Kaiser Permanente Bernard J. Tyson School of Medicine, Pasadena, CA USA

**Keywords:** Adolescents, Family-based treatment, Higher level of care, Hospitalization, Health disparity

## Abstract

**Background:**

Outpatient family-based treatment (FBT) is effective in treating restrictive eating disorders among adolescents. However, little is known about whether FBT reduces higher level of care (HLOC) utilization or if utilization of HLOC is associated with patient characteristics. This study examined associations between utilization of eating disorder related care (HLOC and outpatient treatment) and reported adherence to FBT and patient characteristics in a large integrated health system.

**Methods:**

This retrospective cohort study examined 4101 adolescents who received care for restrictive eating disorders at Kaiser Permanente Northern California. A survey was sent to each medical center to identify treatment teams as high FBT adherence (hFBT) and low FBT adherence (lFBT). Outpatient medical and psychiatry encounters and HLOC, including medical hospitalizations and higher-level psychiatric care as well as patient characteristics were extracted from the EHR and examined over 12 months post-index.

**Results:**

2111 and 1990 adolescents were treated in the hFBT and lFBT, respectively. After adjusting for age, sex, race/ethnicity, initial percent median BMI, and comorbid mental health diagnoses, there were no differences in HLOC or outpatient utilization between hFBT and lFBT. Females had higher odds of any utilization compared with males. Compared to White adolescents, Latinos/Hispanics had lower odds of HLOC utilization. Asian, Black, and Latino/Hispanic adolescents had lower odds of psychiatric outpatient care than Whites.

**Conclusions:**

Reported FBT adherence was not associated with HLOC utilization in this sample. However, significant disparities across patient characteristics were found in the utilization of psychiatric care for eating disorders. More efforts are needed to understand treatment pathways that are accessible and effective for all populations with eating disorders.

**Supplementary Information:**

The online version contains supplementary material available at 10.1186/s40337-024-00976-3.

## Abstract (plain language summary)

Adolescents with restrictive eating treated by Family-Based Treatment (FBT) teams had better early weight gain but no differences in the use of intensive outpatient, residential, partial hospital programs or inpatient psychiatry care when compared to those treated by teams with a low adherence to the FBT approach. Factors such as sex, race, ethnicity, mood disorders, and suicidality were associated with the use of psychiatric services. These findings are consistent with previously documented systematic disparities in accessing psychiatric services across patient demographics and should be used to inform the development of proposed care models that are more inclusive and accessible to all patients.

**Supplementary Information:**

The online version contains supplementary material available at 10.1186/s40337-024-00976-3.

## Introduction

Eating disorders are among the most lethal psychiatric disorders and have a significant impact on an individual’s medical and mental health, conferring substantial societal, healthcare, and socioeconomic costs [9]. Outpatient Family-based Treatment (FBT) is the gold standard treatment modality for most pediatric and adolescent eating disorders [20, 23, 32]. The effectiveness of FBT lies in the fact that it addresses the eating disorder from both biological and psychological/behavioral standpoints. It recognizes that early weight gain is key to reversing medical instability (e.g., bradycardia and hypoglycemia) and the sequelae of malnutrition (e.g., secondary amenorrhea), and that weight restoration is a prerequisite for disease remission [17, 19, 24]. FBT has proven effective in both randomized controlled [20, 23, 32] and clinical settings [1, 4, 12, 20, 37].

Less is known about how outpatient FBT influences the utilization of higher level of care (HLOC), including medical hospitalizations, to reverse medical instability and intensive outpatient, partial hospital, and residential and inpatient psychiatric programs that focus on psychological interventions. The use of FBT has been associated with patients spending fewer days in the hospital and lower treatment costs per patient in remission in randomized controlled studies [3, 16, 22]. Implementing a FBT outpatient program has reduced medical hospitalization of patients with *Anorexia Nervosa* and *Eating Disorder, Not Otherwise Specified* by 56% [16]. In a cohort of patients treated with manualized FBT in the UK, only 27% of patients needed psychiatric HLOC [37]. The main reason for HLOC use was the lack of early weight gain at 3 months [37]. While family and patient factors may mediate or moderate treatment outcomes [13, 14], the key predictor for FBT failure is any history of eating disorder treatment prior to receiving FBT treatment [6]. This suggests the importance of implementing the proper treatment from the start, as the initial treatment may affect the course of subsequent treatment episodes, and ultimately influence clinical outcomes. However, no published data have examined the effect of FBT on medical hospitalization or psychiatric HLOC utilization rates among children/adolescents at a population level with the consideration of demographic factors such as race, ethnicity, age, and sex. A previous study conducted by this group examined hospitalization rates of 4883 adolescents aged 8–18 with any eating disorder diagnosis in 2015–2019 and found that 5.4% of adolescents had medical hospitalizations during the 12-month period after diagnosis [18]. Hospitalization rates differed by ethnicity, with Latino/Hispanic adolescents less likely to be hospitalized than White adolescents, but there were no differences by age, sex, or insurance status.

A better understanding of whether outpatient FBT reduces the utilization of HLOC is essential on multiple fronts. While it is necessary for a child/adolescent to receive treatment, sending a child/adolescent to HLOC can be a significant disruption to a family system, especially for younger children. HLOC may not be a viable option for families due to costs or if HLOC facilities are not accessible in their communities. Also, HLOCs are much more labor-intensive and costly. Knowing whether FBT can minimize HLOC utilization in controlled clinical and research settings and at a population level can help clarify the care pathways and expectations for treatment teams and families. It can also assist healthcare policymakers in program planning to ensure treatment pathways are accessible, inclusive, and culturally appropriate for children/adolescents from diverse socioeconomic and demographic backgrounds.

This is a retrospective cohort study comparing utilization of HLOC, which includes medical hospitalizations and psychiatric intensive outpatient, partial hospital, and residential and inpatient psychiatric programs and outpatient utilization (medical and psychiatric) over 12 months post initial restrictive eating disorder diagnosis by adherence to FBT based on the outpatient treatment teams’ self-report. Evidence shows that FBT is effective in facilitating early weight gain, and that early weight gain reduces HLOC use, therefore, we hypothesize that patients initially seen by treatment teams with a high level of adherence to FBT (hFBT) will have lower utilization of HLOC than those with teams with low adherence to FBT (lFBT). We also hypothesize disparities in utilizing HLOC based on sex, race, and ethnicity.

## Methods

### Study participants

This retrospective cohort study included 4101 adolescents who received care for restrictive disordered eating and eating disorders in Kaiser Permanente Northern California (KPNC). KPNC is a non-profit integrated healthcare delivery system serving a highly diverse membership of over 4.5 million (approximately a third of the region's population) with over 500,000+ adolescent (aged 11–18) members. Its membership is highly representative of other insured populations [7]. Substance use and psychiatry treatment services are covered benefits, provided internally for most KPNC members. KPNC provides comprehensive pediatric eating disorder treatment, including outpatient medical and mental health services (outpatient and intensive outpatient programs) and inpatient medical services. Contracted facilities provide higher-level mental health services, including partial hospital, residential, and inpatient psychiatric treatment.

The cohort included all KPNC adolescents aged 8–18 who had at least one office-based visit with a diagnosis of restrictive eating disorder or a diagnosis that was suggestive of restrictive eating between 1/2015 and 4/2021. Eligible diagnoses included anorexia nervosa (ICD9: 307.1; ICD10: F50.00, F50.01, F50.02), avoidant/restrictive food intake disorder (F50.82), unspecified eating disorder (ICD9: 307.59, 307.50; ICD10: F50.8, F50.89, F50.9) where the patient’s weight at diagnosis was lower than the most recent previously recorded weight in the medical record and malnutrition (ICD9: 261, 262, 263.0, 263.1, 263.2, 263.8, 263.9, 458.0, 783.0, 783.21, 783.9; ICD10: E43, E44.0, E44.1, E45, E46, I95.1, R00.1, R63.0, R63.4, R63.8, Z72.4) or inappropriate diet and eating habits (ICD9: V69.1) with a diagnosis of abnormal weight loss (ICD9: 783.2; ICD10: R63.4). Adolescents with a prior eating disorder-related diagnosis or more than 60 days in lapse of KPNC membership during the study period were excluded.

## Procedures and measures

### Survey data

Online surveys were sent to the Pediatric chiefs and Child/Adolescent Psychiatry managers across all medical centers in the KPNC region (n = 15 medical centers). The Pediatric chiefs and child/adolescent psychiatry managers collaborated with the treating providers within their clinics to ensure accurate responses. These surveys were used to identify best practices of early phase pediatric eating disorder care by assessing eating disorder clinician training, clinical resources and capacity, and services provided at each medical center (see Additional file 1: Table S1 for survey questions).

### Electronic health record (EHR) data: covariates

Clinical and demographic characteristics, including patient sex, age, race/ethnicity, insurance coverage status at index (Medicaid vs. other), index eating disorder diagnosis, and comorbid mental health conditions including mood disorders, anxiety disorders, substance use disorders, and the presence of suicidal ideations in the year prior to the index date were extracted from the EHR (see Additional file 2: Table S2 for diagnosis codes). Weight and body mass index (BMI) at index, and at 60 ± 30-days, 183 ± 30-days and 365 ± 30-days post index were also extracted from the EHR. BMI was used to calculate the percent median BMI (%mBMI), which is commonly used in pediatric eating disorder research as a proxy for the level of malnutrition and is BMI expressed as a percent of the 50th percentile BMI for age and sex [33].

### Outcomes

Inpatient and outpatient medical and psychiatric encounters with a corresponding restrictive eating disorder-related diagnosis were extracted from the EHR. The index date was the first encounter with an eating disorder-related diagnosis during the study period as detailed above. Eating disorder-related utilization over 12 months post index was categorized into four categories: (1) inpatient medical encounters with an eating disorder-related principle diagnosis; (2) higher-level psychiatric encounters including intensive outpatient, residential, partial hospital programs or inpatient psychiatry provided both inside the health plan as well as encounters covered by KPNC but delivered outside of the health plan; (3) outpatient medical (primary care, family medicine); and (4) outpatient psychiatry. All encounters had an associated restrictive eating disorder diagnosis.

### Analysis

Appropriate bivariate analyses (e.g., chi-square, *t* tests, etc.) examined differences between FBT adherence groups and patient characteristics. Weight change was examined between baseline and 60-, 183- and 365-days post index allowing for ± 30-day window around the follow-up time point. Although we allowed for a ± 30-day window around the follow-up time point, there was missing data. To address missing weight data, we implemented multiple imputation methods with 30 iterations using PROC MI and PROC MIANALYZE in SAS [38, 40, 41]. Both reported values and imputed values for patient weight change were reported. Linear regression models with a normal distribution were used to examine weight change post index separately for each follow-up time point.

Dichotomous indicators were created for each utilization type (outpatient medical, outpatient psychiatry, medical hospitalizations, higher-level psychiatry) as well as combined measures for any outpatient eating disorder treatment (e.g., outpatient medical and/or psychiatric encounters) and higher levels of care (HLOC) defined as eating disorder related medical hospitalizations and/or higher-level psychiatry.

Logistic regression analyses examined associations between these dichotomous outcomes and the FBT adherence groups. All models adjusted for age, sex, race/ethnicity, %mBMI at index, and comorbid mental health conditions (e.g., mood disorders, anxiety disorders, substance use disorders and the presence of suicide ideations).

## Results

Out of the 15 medical centers, 67% (n = 10) indicated that they had a MD as part of the eating disorder treatment team, 80% (n = 12) had a therapist and 60% (n = 9) had a dietician. Forty-six percent (n = 7) said that “FBT [was] being delivered to all patients as the first-line treatment, including the explanation of the phases of treatment, agnostic approach, parent refeeding and emphasis of medical recovery (e.g., discuss weight gain goals)” (3A from Additional file 1: Table S1) while 40% (n = 6) said that their “overall approach [was] FBT-informed” (3A from Additional file 1: Table S1) and 13% (n = 2) used another modality. Twenty-six percent of the medical centers (n = 4) noted that a provider on the care team had attended a post-doctoral program focused on eating disorders, 13.3% (n = 2) of medical centers noted that providers on the care team attended a certificate program from the International Association of Eating Disorder Professionals or the Training Institute for Child and Adolescent Eating Disorders, 67% (n = 10) of medical centers had providers on the care team who attended continuing medical education sessions related to treatment for pediatric eating disorders and 7% (n = 1) had a provider on the care team who attended the Adolescent Medicine Fellowship Program accredited by the Accreditation Council for Graduate Medical Education. Out of the 15 medical centers, 4 stated that care providers did not have any specialized training.

Medical centers were classified as having high adherence to FBT if: (1) the medical center had a MD (question 2a from Additional file 1: Table S1) and therapist (2c from Additional file 1: Table S1) who treated eating disorders among adolescents within the medical center and (2) FBT was being delivered to all patients as the first-line of treatment (question 3a from Additional file 1: Table S1). Using these criteria, 5 out of 15 medical centers qualified as having high adherence to the FBT approach (hFBT) and 10 medical centers were considered not to have high adherence to the FBT approach (i.e., low adherence; lFBT). The number of adolescents with an eating disorder diagnosis during the study time period (1/2015–4/2021) was similar across both groups (hFBT n = 2111 [51.5%], lFBT n = 1990 [48.5%]; *p* = 0.059).

There were no significant differences in patient sex or age between the FBT adherence groups. Compared to adolescents in the hFBT group, the lFBT group had more Asian (15.0% vs. 7.7%) and Latino/Hispanic (34.5% vs. 20.9%) and fewer White (33.8% vs. 55.1%) adolescents (*p* < 0.001). Those in the lFBT group had lower %mBMI at the index visit (99.9% vs. 103.3%) than those in the hFBT group (*p* < 0.001). The lFBT group had more missing weight data compared to patients in the hFBT group (34% vs. 30% at 30 days post index, *p* < 0.01; 52% vs. 46% at 183 days post index, *p* < 0.01; 63% vs. 60% at 365 days post index, *p* = 0.7). Those in the lFBT group had less weight gain in the first 2 months of treatment than the hFBT group (complete cases: 2.1 ± 8.9 pounds lFBT group vs. 3.3 ± 8.8 pounds hFBT, *p* < 0.001; imputed data: 2.0 ± 8.8 pounds lFBT vs. 3.1 ± 8.7 pounds hFBT, *p* < 0.001). There were no significant differences in Medicaid coverage or prevalence of comorbid mood, anxiety, substance use or suicidality diagnoses between groups. Seventy-three percent of adolescents had at least one eating disorder related encounter in the year post index (72.9% hFBT vs. 72.3% lFBT; *p* = 0.696). There were no differences between groups in prevalence of any of the utilization measures examined (Table 1).

**Table 1:** Patient demographics, utilization and weight gain by family based treatment (FBT) adherence group (n = 4101)

	High FBT adherence group (n = 2111)	Low FBT adherence group (n = 1990)	*p* value
Female—n (%)	1628 (77.1)	1575 (79.2)	0.117
Age—mean (SD)	14.7 (2.4)	14.8 (2.3)	0.085
*Race/ethnicity*			
Asian	163 (7.7)	299 (15.0)	< .001
Black	77 (3.7)	70 (3.5)	
Latino/Hispanic, any race	442 (20.9)	686 (34.5)	
Multiracial	156 (7.4)	162 (8.1)	
Native American/Alaskan Native	5 (0.2)	3 (0.2)	
Native Hawaiian/Pacific Islander	7 (0.3)	9 (0.5)	
Unknown	97 (4.6)	89 (4.5)	
White, non-Hispanic	1164 (55.1)	672 (33.8)	
%Median BMI at index—mean (SD)	103.3 (23.1)	99.9 (20.7)	< .001
Medicaid coverage at index—n(%)	350 (16.6)	319 (16.0)	0.634
*Weight gain—mean (SD)*			
Complete Cases +			
60 ± 30 days post index	3.3 (8.8)	2.1 (8.9)	< .001
183 ± 30 days post index	7.1 (11.9)	6.3 (12.0)	0.095
365 ± 30 days post index	10.7 (14.7)	9.4 (14.9)	0.079
Imputed data+			
60 ± 30 days post index	3.1 (8.7)	2.0 (8.8)	< .001
183 ± 30 days post index	6.7 (11.5)	5.8 (11.7)	0.047
365 ± 30 days post index	10.2 (14.3)	9.4 (14.4)	0.145
*Diagnoses 12 M prior to index—n (%)*			
Mood	634 (30.0)	621 (31.2)	0.415
Substance use	82 (3.9)	90 (4.5)	0.308
Anxiety	110 (5.2)	106 (5.3)	0.868
Suicidality	129 (6.1)	122 (6.1)	0.979
*Any HLOC*^a^* utilization 1 year post index -n (%)*	1538(72.9)	1439(72.3)	0.696
Outpatient	1535 (72.7)	1439 (72.3)	0.773
Medical outpatient	1275 (60.4)	1162 (58.4)	0.191
Psychiatry outpatient	1051 (49.8)	953 (47.9)	0.224
Higher levels of care	203 (9.6)	179 (9.0)	0.494
Medical hospitalizations	108 (5.1)	106 (5.3)	0.762
Higher level psychiatric care	136 (6.4)	130 (6.5)	0.907

^a^*HLOC* higher level of care (HLOC) of treatment, including medical hospitalizations to reverse medical instability and intensive outpatient, partial hospital, and residential and inpatient psychiatric programs specific that focuses on psychological interventions

After adjusting for age, sex, race/ethnicity, %mBMI at index, and comorbid mental health diagnoses, there were no differences between hFBT and lFBT in any of the utilization categories examined. Females had higher odds of utilization than males across all types of utilization (e.g., any outpatient [Adjusted Odds Ratio[AOR] = 1.80, 95% Confidence Interval [CI]  1.53–2.12], outpatient medical [AOR = 1.59, 95% CI 1.36–1.86], outpatient psychiatry [AOR = 1.70, 95% CI 1.45–1.98] and any HLOC [AOR 1.74, 95% CI 1.29–2.34], hospitalizations [AOR = 1.68, 95% CI 1.15–2.44], and higher-level psychiatry encounters [AOR = 1.59, 95% CI 1.12–2.27]). Older adolescents had higher odds of any outpatient (AOR = 1.07, 95% CI 1.04–1.10), and outpatient medical (AOR = 1.05, 95% CI 1.02–1.08) utilization. Adolescents with a higher %mBMI at index had lower odds of any outpatient (AOR = 0.99, 95% CI 0.99–0.99), outpatient medical (AOR = 0.99, 95% CI 0.98–0.99), any HLOC (AOR 0.97, 0.97–0.98), medical hospitalizations (AOR = 0.97, 95% CI 0.96–0.98) and higher-level psychiatry (AOR = 0.98, 95% CI 0.97–0.98). Compared to White adolescents, Latino/Hispanic adolescents had lower odds of HLOC utilization (AOR 0.64, 0.48–0.85, *p* < 0.001) and higher-level psychiatry (AOR = 0.64, 95% CI 0.45–0.89). Asian, Black and Latino/Hispanic adolescents had lower odds of any outpatient care (Asian: AOR = 0.74, 95% CI 0.59–0.94; Black: AOR = 0.58, 95% CI 0.41–0.83, Latino/Hispanic: AOR = 0.78, 95% CI 0.66–0.96) and outpatient psychiatry (Asian: AOR = 0.71, 95% CI 0.57–0.87; Black: AOR = 0.53, 95% CI 0.37–0.76, Latino/Hispanic: AOR = 0.72, 95% CI 0.62–0.85) compared with White adolescents. Latino/Hispanic patients also had lower odds of higher levels of care (AOR = 0.66, 95% CI 0.49–0.88). Adolescents with a prior mood diagnosis had lower odds of outpatient medical (AOR = 0.69, 95% CI 0.59–0.80), HLOC (AOR = 0.68, 95% CI 0.51–0.91) and medical hospitalizations (AOR = 0.41, 95% CI 0.27–0.65) and higher odds of outpatient psychiatry (AOR = 1.41, 95% CI 1.21–1.64) compared to those without a prior mood diagnosis. Adolescents with a comorbid anxiety diagnosis also had higher odds of outpatient psychiatry (AOR = 1.40, 95% CI 1.05–1.87) compared to those without a diagnosis. A prior substance use diagnosis was associated with lower odds of any outpatient (AOR = 0.63, 95% CI 0.45–0.90) and outpatient medical (AOR = 0.62, 95% CI 0.45–0.86) encounters while those with a suicidality diagnosis had higher odds of HLOC (AOR = 1.91, 95% CI 1.19–3.07) and higher-level psychiatry (AOR = 2.25, 95% CI 1.36–3.71) compared to those without a suicidality diagnosis. Patients with Medicaid coverage had lower odds of outpatient psychiatric utilization (AOR = 1.27, 95% CI 1.06–1.51) (Table 2).

**Table 2:** Logistic regression models examining utilization over 12 months post index among patients with a restrictive eating disorder (n = 4101)

	Outpatient		Outpatient—medical only		Outpatient—psychiatry only		Higher levels of care (HLOC)		HLOC—hospitalization		HLOC—higher level of psychiatry	
	aOR (95% CI)	*p* value	aOR (95% CI)	*p* value	aOR (95% CI)	*p* value	aOR(95% CI)	*p* value	aOR (95% CI)	*p* value	aOR (95% CI)	*p* value
Intercept	1.79 (1.00–3.20)	0.050	2.55 (1.46–4.44)	0.001	0.58 (0.33–0.99)	0.047	1.52 (0.54–4.28)	0.432	3.30 (0.86–12.67)	0.082	0.53 (0.15–1.81)	0.310
Medical center with high adherence to FBT (vs. low adherence)	0.96 (0.83–1.10)	0.541	1.04 (0.91–1.19)	0.542	1.02 (0.89–1.16)	0.805	0.94 (0.75–1.17)	0.563	0.84 (0.63–1.12)	0.237	0.88 (0.68–1.13)	0.315
Female (vs. male)	1.80 (1.53–2.12)	< .001	1.59 (1.36–1.86)	< .001	1.70 (1.45–1.98)	< .001	1.74 (1.29–2.34)	< .001	1.68 (1.15–2.44)	0.007	1.59 (1.12–2.27)	0.009
Age at index	1.07 (1.04–1.10)	< .001	1.05 (1.02–1.08)	0.001	1.01 (0.99–1.04)	0.343	0.97 (0.93–1.02)	0.261	0.94 (0.89–1.00)	0.055	0.98 (0.93–1.04)	0.522
Race/ethnicity (ref: white)												
Asian	0.74 (0.59–0.94)	0.014	0.97 (0.78–1.21)	0.786	0.70 (0.57–0.87)	0.001	0.72 (0.50–1.02)	0.068	0.83 (0.53–1.29)	0.407	0.79 (0.52–1.19)	0.255
Black	0.58 (0.41–0.84)	0.003	0.71 (0.50–1.02)	0.061	0.53 (0.37–0.76)	< .001	0.54 (0.25–1.18)	0.122	0.72 (0.29–1.83)	0.495	0.34 (0.11–1.10)	0.071
Hispanic, any race	0.78 (0.66–0.93)	0.006	0.96 (0.82–1.13)	0.623	0.72 (0.62–0.85)	< .001	0.66 (0.49–0.88)	0.005	0.74 (0.51–1.08)	0.117	0.67 (0.48–0.95)	0.024
Other^a^	0.86 (0.69–1.08)	0.200	0.93 (0.76–1.14)	0.511	0.81 (0.67–0.99)	0.040	0.96 (0.69–1.32)	0.782	0.72 (0.45–1.15)	0.166	1.04 (0.72–1.51)	0.835
%mBMI at index	0.99 (0.99–0.99)	< .001	0.99 (0.98–0.99)	< .001	1.00 (0.99–1.00)	0.130	0.97 (0.97–0.98)	< .001	0.97 (0.96–0.98)	< .001	0.98 (0.97–0.99)	< .001
Diagnoses in year prior to inde												
Mood	0.94 (0.79–1.11)	0.457	0.69 (0.59–0.80)	< .001	1.41 (1.21–1.64)	< .001	0.68 (0.51–0.91)	0.009	0.41 (0.27–0.65)	< .001	0.83 (0.60–1.15)	0.262
Anxiety	1.20 (0.86–1.69)	0.281	0.95 (0.71–1.27)	0.717	1.40 (1.05–1.87)	0.023	0.99 (0.61–1.61)	0.964	0.74 (0.34–1.62)	0.446	1.19 (0.71–2.00)	0.515
Substance use	0.63 (0.45–0.90)	0.010	0.62 (0.45–0.86)	0.004	0.77 (0.55–1.07)	0.116	1.04 (0.60–1.81)	0.886	1.18 (0.54–2.57)	0.670	1.04 (0.57–1.92)	0.893
Suicidality	1.04 (0.75–1.43)	0.832	1.21 (0.90–1.62)	0.199	0.80 (0.60–1.06)	0.125	1.91 (1.19–3.07)	0.007	1.75 (0.82–3.75)	0.150	2.25 (1.36–3.71)	0.002
Medicare	0.98 (0.81–1.19)	0.848	1.14 (0.95–1.36)	0.154	0.79 (0.66–0.94)	0.008	0.87 (0.63–1.21)	0.408	1.02 (0.68–1.55)	0.908	0.69 (0.46–1.05)	0.083

^a^Other race was created due to small n's and includes Native American/Alaskan Native, Native Hawaiian/Pacific Islander, Multiracial and Unknown race; *%mBMI* percent median body mass index, *aOR* adjusted odds ratio, *95% CI* 95% confidence interval

Linear regression models examining weight change post index separately for each follow-up time point found that patients receiving treatment in hFBT had more weight gain than those in low adherence medical centers at 2 months post index (estimate = 0.62, standard error = 0.31, *p* = 0.045); there were no significant differences at the other time points. Hispanic patients lost weight between the index visit and the 2-month follow-up compared with White patients (estimate = − 0.97, *p* value = 0.010), as did female patients compared with males (estimate = − 0.83, standard error = 0.39, *p* = 0.033). There were no other differences in weight change across race/ethnicity or sex at the other time points. Older patients lost weight compared to younger patients at the 6-month (estimate = − 0.22, standard error = 0.11, *p* = 0.046) and 12-month (estimate = − 0.80, standard error = 0.15, *p* < 0.001) follow-ups (Table 3).

**Table 3:** Linear regression models examining weight gain post index among patients with restrictive eating disorder (n = 4101)

	2 months post index^a^			6 months post index^a^			12 months post index^a^		
	Estimate	Std err	*p* value	Estimate	Std err	*p* value	Estimate	Std err	*p* value
Intercept	12.20	1.17	< .001	22.57	1.70	< .001	34.16	2.55	< .001
Medical center with High adherence to FBT (vs. low adherence)	0.62	0.31	0.045	0.24	0.40	0.540	0.16	0.61	0.796
Female (vs. male)	− 0.83	0.39	0.033	− 0.99	0.60	0.099	− 1.46	0.82	0.078
Age at index	− 0.05	0.07	0.503	− 0.22	0.11	0.046	− 0.80	0.15	< .001
Race/ethnicity (ref: white)									
Asian	0.09	0.54	0.867	− 0.45	0.74	0.544	0.53	1.12	0.639
Black	− 0.34	0.88	0.701	− 0.84	1.32	0.527	1.28	2.07	0.538
Hispanic, any race	− 0.97	0.38	0.010	− 0.91	0.53	0.086	− 0.58	0.77	0.449
Other^b^	− 0.37	0.48	0.449	− 0.55	0.66	0.404	0.06	0.98	0.947
%mBMI at index	− 0.42	0.04	< .001	− 0.61	0.05	< .001	− 0.58	0.07	< .001
Diagnoses in year prior to index									
Mood	0.28	0.38	0.465	0.84	0.54	0.118	− 0.19	0.81	0.815
Anxiety	0.43	0.68	0.531	− 0.98	1.06	0.356	− 1.04	1.26	0.411
Substance use	1.08	0.83	0.193	− 1.11	1.16	0.342	− 2.36	1.58	0.137
Suicidality	1.07	0.66	0.106	3.92	0.96	< .001	5.71	1.36	< .001

Models using only complete cases had the same results—2M: estimate = 0.8463, *p* value = 0.0088; 6M and 12M ns

^a^Follow-up windows included a ± 30 day window; e.g., 2 months post index = 60 ± 30d post index, 6 months = 183 ± 30d post index, 12 months = 365 ± 30d post index

^b^Other race was created due to small n's, includes Native American/Alaskan Native, Native Hawaiian/Pacific Islander, Multiracial and Unknown race

## Discussion

Our study did not find differences in HLOC utilization for eating disorders between medical centers reporting high adherence to FBT compared to those reporting low adherence to FBT. However, we did find that, as expected, adolescents who received initial treatment from medical centers reporting hFBT had more weight gain in the first 2 months than those initially treated in medical centers reporting lFBT. Still, the early weight gain did not translate into lower utilization of HLOC between groups. This is an unexpected result because, logically, if we have an outpatient treatment modality that has proven to be effective, we expect patients to improve in the outpatient setting and utilize less of the higher-intensity care. Our results illustrate the complexity of implementing an effective treatment modality in the real world. Many factors are at play when we deliver a treatment modality in a large health system that involves a multi-disciplinary approach, including patient characteristics, provider training/fidelity, and practice support (e.g., staffing, team-based/collaboration models). Our results may suggest that only a subset of pediatric patients with restrictive eating disorders will benefit from an outpatient FBT-only approach and astute providers are referring patients to HLOC when it is necessary. Or there could be provider and system issues, including provider training, lack of fidelity, staffing shortages, and coordination challenges even in the hFBT group, where the treatment helped early weight gain, but the use of HLOC was still necessary for patients to sustain weight gain and achieve recovery. In future studies, it is recommended to include some of the abovementioned components, such as gaining a better understanding of patient characteristics that can benefit from an outpatient FBT-only approach versus those who may require a higher level of care (HLOC). Additionally, future work is needed to refine the methods to measure a treatment team's FBT fidelity and their practice support system.

We found significant disparities in patient characteristics among patients accessing psychiatric services for eating disorders. Females had higher odds of utilizing medical and mental health services than males in all treatment settings. This disparity in the use of eating disorder mental health services is consistent with existing literature on treatment for other mental health conditions where female adults and adolescents utilize more mental health services than their male counterparts [31, 39] regardless of the treatment setting. The difference in medical visit utilization for eating disorders in male adolescents may stem from the challenges of identifying these disorders in boys. Parents and pediatricians may not notice the signs of eating disorders in males since they present differently than in females. For example, parents may mistake boys getting thinner while growing taller as a normal part of puberty. Pediatricians may overlook disordered eating behavior in boys if they only look for dietary changes related to intentional weight loss without considering the desire for a specific body shape or muscularity. Furthermore, medical guidelines are mainly based on research and clinical experience with female patients, which may not provide enough guidance for clinicians to screen and treat boys properly [28].

We also found a disparity in the utilization of outpatient eating disorder care among Asian, Black, and Latino/Hispanic when compared to White adolescents. When medical and psychiatry visits were evaluated separately, it became clear that the disparity was mainly explained by Asian, Black, and Latino/Hispanic adolescents having lower odds of outpatient psychiatry visits. The disparity persisted for Latinos/Hispanics for psychiatric HLOC but not Asians and Blacks. Racial and ethnic differences among non-white populations accessing mental health services have been widely reported among adult and adolescent populations [15, 27, 36]. It is possible that our current treatment and referral system may not have adequately adapted evidence-based treatment considering the patient and family’s cultural context and values vital to engaging Latino/Hispanic patients [34]. In addition, the health insurance landscape has become very complex in the United States, with costs shifted to patients using high cost-sharing plans (e.g., high deductible, copayment, coinsurance, and out-of-pocket limits) [29]. Patients may have higher rates of high deductibles and copayment insurance plans, thus influencing their decisions for HLOC.

The racial and ethnic disparity found in accessing outpatient psychiatric visits but not in medical visits suggested families are comfortable seeking care for their child’s physical symptoms and sequelae of malnutrition but perhaps not as comfortable accepting mental health services with the same ease. This disparity could be associated with factors previously explored as barriers for minorities to access mental health services, including mental health stigma, the lack of understanding or having different views of mental health treatment, the overwhelmingly White mental health workforce, or prior treatment experiences of being discriminated against [5, 10, 25, 26, 30]. In addition, engaging in FBT in our system typically requires weekly-biweekly visits with medical providers, mental health providers, and dietitians separately. There are natural and practical considerations for families to be able to engage in treatment fully which include costs, transportation, and time (e.g., time to care for other children and forgone work time to participate in appointments). While clinicians may master cultural competency and skills to engage families from diverse backgrounds, more drastic and urgent changes in our treatment system are needed to reduce barriers for our minority adolescents and families to engage in treatment. These strategies include having integrated mental health/behavioral models, where FBT therapists are practicing alongside pediatricians in the medical setting to reduce the number of appointments, or implementing innovative models, such as the FBT-Primary Care [21] and FBT-Home base [8, 11] models, where the former trains pediatricians to deliver FBT concepts and the latter trains community therapists to deliver FBT in the home setting.

In our cohort, the lFBT group was more racial and ethnically diverse and sicker (lower %mBMI) than the hFBT group at the beginning of treatment. We compared the cohort’s demographic composition to the age-matched pediatric population within KPNC and found no difference between our groups and the corresponding larger population (data not shown). The difference in race/ethnicity composition and severity between the lFBT and hFBT groups suggest at least two areas for further exploration: (1) Do FBT-trained therapists tend to practice in locations with a higher proportion of White adolescents? Moreover, (2) How adaptable is FBT among non-White populations? For the first question, the current study cohort is from a “pre-pandemic” period when the use of telehealth was limited. It will be important to re-evaluate this question with “post-pandemic” data to examine the impact of telehealth and whether this disparity still exists now that virtual therapists can practice anywhere, and patients have in-person and telehealth options. Centralized, virtual models of FBT care could help ameliorate some of these disparities. The second question has been partly explored among adolescents insured by public insurance [2] and community-based settings [8, 11], which tend to have adolescents from more diverse racial and ethnic backgrounds. FBT was feasible, acceptable, and effective in these settings [2, 8, 11]. However, these models have only been tested and implemented in small clinic samples. More research is needed to understand the scalability of these models and their effects on addressing health inequities.

Patients with a comorbid mood disorder diagnosis had higher odds of outpatient psychiatry but lower odds of HLOC, which may shed light on the course of treatment of adolescents with comorbid mood and eating disorders. It is possible that treating their mood disorder also helped with their eating disorder, or there may be some overlap of treatment where patients are receiving eating disorder and depression treatment concurrently. The finding that adolescents with suicidal ideations in this cohort had higher odds of psychiatric HLOC is expected as active suicidal ideations is a mental health crisis requiring inpatient interventions.

### Strengths and limitations

This study provides a bird’s eye view of pediatric eating disorder care in a large insured population where entry to care is typically through the medical setting. It adds to our current understanding of the effectiveness of FBT in terms of early weight gain and its effect on the use of HLOC. It offers real-world utilization of eating disorder-related care among a diverse patient population. The interpretation of findings is limited by the following: Inpatient utilization was low (~ 5%) which may have made it harder to detect significant differences. We included a heterogeneous cohort by extracting EHR data using ICD-9 and ICD-10 diagnoses associated with eating disorders, therefore, our cohort likely includes subthreshold restrictive eating disorders. Nevertheless, we only included adolescents with a history of restrictive eating disorder diagnoses or weight loss and other eating disorder diagnoses, assuming that some level of weight restoration needed to happen and FBT could be beneficial. Adherence to FBT is based on self-report by the Pediatric chiefs and/or Child/Adolescent Psychiatry managers or their designees and may or may not correlate with fidelity. There may be an overlap in the treatment approaches between hFBT and lFBT groups. However, this resembles mental health care delivered in the community where treatment modality and program descriptions are based on what providers report on their program materials (e.g. website and brochures). To better correlate FBT adherence to fidelity, future studies should consider including FBT certification and whether providers deliver key principles of FBT. More efforts are needed to make FBT training and certification more accessible and to refine population-based measures to assess FBT adherence. The survey answers are likely reflecting the collective impression of the treatment being delivered at the time of the survey, which may or may not have factored in the changes in practice during the study period that may affect FBT adherence, including staff turnover, staff training, and pandemic service disruptions. Lastly, the hFBT and lFBT group designations are based on the location of the initial visit. While it is possible that we did not account for the location of their subsequent care (e.g., a family might have moved from an hFBT to an lFBT location during the study period), we expect the number of patients to be small because members in our health system usually receive care from the medical center close to their home.

## Conclusion

Our findings suggest that the impact of FBT on HLOC utilization is uncertain. However, there are significant differences in early weight gain and HLOC utilization depending on sex, race, and ethnicity. These differences suggest that the effectiveness of FBT may be affected by systemic inequalities in the availability of eating disorder and mental health services for males and racial and ethnic minorities. Though we found that patients in the high FBT adherence group had early weight gain suggesting FBT to be an effective modality for restrictive eating behaviors and disorders treatment, we did not find significant differences in psychiatric HLOC utilization in this large clinical cohort. We found utilization of psychiatric HLOC to be primarily associated with the sex, race, and ethnicity of the adolescents and the presence of co-occurring mood disorders or suicidal ideation. To fully realize the benefits of FBT for adolescents from all demographic backgrounds, it calls for more efforts on strategies to make FBT more accessible and adaptable to minority populations, including strengthening the delivery of FBT in the medical and community practice settings.

### Supplementary Information

**Additional file 1**. Identifying Best Practices of Early Phase Pediatric Eating Disorder Care Survey Administered to Pediatric Chiefs and Child/Adolescent Psychiatry Managers to Determine Adherence to Family Based Treatment.

**Additional file 2**. List of comorbid mental health condition diagnosis codes.

## Data Availability

Research data are not shared.

## Abbreviations

KPNC: Kaiser Permanente Northern California
HLOC: Higher levels of care
FBT: Family-based treatment
EHR: Electronic health record
hFBT: High adherence to family-based treatment
lFBT: Low adherence to family-based treatment
BMI: Body mass index
%mBMI: Percent median body mass index
