# Supplementary material for: Adolescent utilization of eating disorder higher level of care: roles of family-based treatment adherence and demographic factors
Source: J Eat Disord. 2024 Feb 2;12:22. doi: 10.1186/s40337-024-00976-3 (PMC10835916; doi:10.1186/s40337-024-00976-3)
Supplement: Supplementary file 1 — Additional file 1. Identifying Best Practices of Early Phase Pediatric Eating Disorder Care Survey Administered to Pediatric Chiefs and Child/Adolescent Psychiatry Managers to Determine Adherence to Family Based Treatment. [file 40337_2024_976_MOESM1_ESM.docx]

Appendix 1.

Survey: Identifying Best Practices of Early Phase Pediatric Eating Disorder Care

1. Can you tell us your role and the facility where you practice?
   1. Name
   2. Role
   3. Facility (can be more than 1)
2. Can you help us identify the following providers who treat eating disorders in children and adolescents in your medical center?

Please provide the of names for each member of the treatment team:

- 1. MD/NP
  2. Dietitian
  3. Therapist

1. How would you describe your facility’s current approach in treating children and adolescents with eating disorders?
   1. FBT is being delivered to all patients as the first-line treatment, including the explanation of the phases of treatment, agnostic approach, parent refeeding and emphasis of medical recovery (e.g. discuss weight gain goals)
   2. My overall approach is FBT-informed, i.e., I incorporate some FBT concepts as needed based on my clinical judgement
   3. I use modality other than FBT. If yes, specify the modality that you use ______________
   4. Other
2. We would like to offer more continuing education opportunities on pediatric eating disorders in the future. To understand your needs, can you tell us your training/experience in pediatric eating disorders? (Mark all that apply)
   1. Post-doctoral program focused on eating disorders (therapist only)
   2. Certificate program from the International Association of Eating Disorder Professionals or the Training Institute for Child and Adolescent Eating Disorders (therapist, RD and MD/NP)
   3. Adolescent Medicine Fellowship Program that is accredited by the Accreditation Council for Graduate Medical Education (ACGME) (MD only)
   4. Continuing Medical Education sessions
   5. No specialized training. I mostly learn from my patients and colleagues (therapist, RD, MD/NP)
   6. Other:
3. What kind of communications or collaborations are in place to promote consistent treatment strategy and messaging as a treatment team? (Check all that apply)
   1. Joint visits by MD/NP +/- RD +/- therapist
   2. Multidisciplinary case discussions/rounds
   3. Communications among treatment providers are on an as needed basis (e.g. phone calls, Teams, CC Charts and Staff Messages)
   4. Other
4. Please tell us any suggestions that you have on building an FBT early intervention program:
