# Supplementary material for: Adolescent utilization of eating disorder higher level of care: roles of family-based treatment adherence and demographic factors
Source: J Eat Disord. 2024 Feb 2;12:22. doi: 10.1186/s40337-024-00976-3 (PMC10835916; doi:10.1186/s40337-024-00976-3)
Supplement: Supplementary file 2 — Additional file 2. List of comorbid mental health condition diagnosis codes. [file 40337_2024_976_MOESM2_ESM.docx]

**Comorbid mental health condition diagnosis codes**

**Anxiety Disorders**

| **ICD Version** | **Code** | **Description** |
| --- | --- | --- |
| 9 | 300* | Anxiety, dissociative and somatoform disorders |
|  | 309.81 | Posttraumatic stress disorder |
|  | 306.3* | Skin disorder arising from mental factors |
|  | 293.84* | Anxiety disorder in conditions classified elsewhere |
|  | 780.95* | Excessive crying of child, adolescent, or adult |
|  | 780.99* | Other general symptoms |
|  | 799.22* | Irritability |
|  | 799.25 | Demoralization and apathy |
| 10 | F4* | Anxiety, dissociative, stress-related, somatoform and other nonpsychotic mental disorders |
|  | F06.4* | Anxiety disorder due to known physiological condition |
|  | R45.2* | Unhappiness |
|  | R45.3* | Demoralization and apathy |
|  | R45.4* | Irritability and anger |
|  | R45.5* | Hostility |
|  | R45.6* | Violent behavior |
|  | R45.7* | State of emotional shock and stress, unspecified |
|  | R45.81 | Low self-esteem |
|  | R45.82 | Worries |
|  | R45.83 | Excessive crying of child, adolescent or adult |
|  | R45.84 | Anhedonia |

**Mood Disorders**

| ICD Version | Code | Description |
| --- | --- | --- |
| 9 | 296* | Episodic mood disorders |
|  | 293.83* | Mood disorder in conditions classified elsewhere |
|  | 298.0* | Depressive type psychosis |
|  | 301.1* | Affective personality disorder |
|  | 311* | Depressive disorder, not elsewhere classified |
|  | 300.4* | Dysthymic disorder |
|  | 625.4* | Premenstrual tension syndromes |
|  | 799.24 | Emotional lability |
| 10 | F3* | Mood affective disorders |
|  | F06.3 | Mood disorder due to known physiological condition |
|  | R45.86 | Emotional lability |

**Suicidality Disorders**

| ICD Version | Code | Description |
| --- | --- | --- |
| 9 | V62.84 | Suicidal ideations |
|  | R45.851 | Suicidal ideations |
|  | E95 | Suicide And Self-Inflicted Injury |
| 10 | T14.91 | Suicide attempt |
|  | X7.1*-X7.9* |  |
|  | X8.0*-X8.3* |  |
|  | T40.0X2 | Poisoning by opium, intentional self-harm |
|  | T40.1X2 | Poisoning by heroin, intentional self-harm |
|  | T40.5X2 | Poisoning by cocaine, intentional self-harm |
|  | T40.7X2 | Poisoning by cannabis, intentional self-harm |
|  | T40.8X2 | Poisoning by lysergide [LSD], intentional self-harm |
|  | T40.902 | Poisoning by unspecified psychodysleptics [hallucinogens], intentional self-harm |
|  | T40.992 | Poisoning by other psychodysleptics [hallucinogens], intentional self-harm |

**Substance Use Disorders**

| ICD Version | Code | Description |
| --- | --- | --- |
| 9 | 291* | Alcohol-induced mental disorders |
|  | 292* | Drug-induced mental disorders |
|  | 303* | Alcohol dependence syndrome |
|  | 304* | Drug dependence |
|  | 305* | Nondependent substance abuse |
|  | 909.0* | Late effect of poisoning due to drug, medicinal or biological substance |
|  | 909.5* | Late effect of adverse effect of drug medicinal or biological substance |
|  | 965.00 | Poisoning by opium (alkaloids), unspecified |
|  | 965.01 | Poisoning by heroin |
|  | 969.6* | Poisoning by psychodysleptics (hallucinogens) |
|  | 970.81 | Poisoning by cocaine |
|  | 995.29 | Unspecified adverse effect of other drug, medicinal and biological substance |
|  | E850.0 | Accidental poisoning by heroin |
|  | E850.2 | Accidental poisoning by other opiates and related narcotics |
|  | E854.1 | Accidental poisoning by psychodysleptics (hallucinogens) |
|  | E929.2 | Late effects of accidental poisoning |
|  | E935.0 | Heroin causing adverse effects in therapeutic use |
|  | E935.2 | Other opiates and related narcotics causing adverse effects in therapeutic use |
|  | E939.6 | Psychodysleptics [hallucinogens] causing adverse effects in therapeutic use |
|  | E939.7 | Psychostimulants causing adverse effects in therapeutic use |
|  | E962.0 | Assault by drugs and medicinal substances |
|  | E980.0 | Poisoning by analgesics, antipyretics, and antirheumatics, undetermined whether accidentally or purposely inflicted |
|  | E980.2 | Poisoning by other sedatives and hypnotics, undetermined whether accidentally or purposely inflicted |
|  | E989 | Late effects of injury, undetermined whether accidentally or purposely inflicted |
| 10 | F1* | Substance related disorders |
|  | F55 | Abuse of non-psychoactive substances |
|  | T40.01-T40.05* | Poisoning by, adverse effect of and underdosing of opium. |
|  | T40.11-T40.15* | Poisoning by and adverse effect of heroin |
|  | T40.51-T40.55* | Poisoning by, adverse effect of and underdosing of cocaine |
|  | T40.71-T40.75* | Poisoning by, adverse effect of and underdosing of cannabis (derivatives) |
|  | T40.81-T40.85* | Poisoning by and adverse effect of lysergide [LSD] |
|  | T40.91-T40.95* | Poisoning by, adverse effect of and underdosing of other and unspecified psychodysleptics |
|  | T40.991-T40.995* | Poisoning by other psychodysleptics [hallucinogens] |
